# Variable response of eastern filbert blight resistance sources in New Jersey

**DOI:** 10.3389/fpls.2024.1419265

**Published:** 2024-07-24

**Authors:** Daniel C. Jacobs, Ronald S. Revord, John M. Capik, Shawn A. Mehlenbacher, Thomas J. Molnar

**Affiliations:** ^1^ Department of Plant Biology, Rutgers University, New Brunswick, NJ, United States; ^2^ Center for Agroforestry, School of Natural Resources, University of Missouri, Columbia, MO, United States; ^3^ Department of Horticulture, Oregon State University, Corvallis, OR, United States

**Keywords:** hazelnut, durable resistance, breakdown, quantitative resistance, ‘Ratoli’

## Abstract

Eastern filbert blight (EFB), caused by *Anisogramma anomala*, is the primary limiting factor for hazelnut (*Corylus* sp.) production in the United States. In this study, 82 cultivars and selections shown to be resistant or tolerant to EFB in Oregon were field planted in New Jersey in 2017 and 2019 and evaluated for their EFB response under high disease pressure. The trees carry known single resistance (R) genes with most mapped to their respective linkage groups (LG), including LG2, LG6, and LG7, or they express quantitative resistance (QR, horizontal resistance). Disease incidence and severity was documented, stem cankers counted and measured, and the proportion of diseased wood calculated. The EFB disease response of some cultivars/selections varied considerably between New Jersey and Oregon while others were consistent. Trends were observed in relation to resistance source origin and LGs, which provide insight into durability and usefulness of resistance. In striking contrast to Oregon, nearly all selections with R-genes mapped to LG6, including those carrying the ‘Gasaway’ resistance allele, exhibited severe EFB infections (232 of 266 [87%]). This finding is of consequence since the U.S. hazelnut industry currently relies solely on LG6 resistance for EFB resistance. Further, for the first time, EFB was observed on several selections carrying LG7 resistance, specifically offspring of ‘Ratoli’ from Spain. Interestingly, selections carrying LG7 resistance from origins other than ‘Ratoli’ remained free of EFB, with one exception, all selections carrying LG2 (n=9) resistance also remained free from EFB. Interestingly, the EFB responses of selections expressing QR (n=26) more closely resembled the disease phenotypes they exhibited in Oregon. Overall, the divergence in EFB response between Oregon and New Jersey, where pathogen populations differ, supports the presence of pathogenic variation in *A. anomala* and highlights potential limitations of using single R-genes to manage the disease. Results also suggest trees expressing QR may be more stable across pathogenic populations.

## Introduction

Eastern filbert blight (EFB) is the primary limiting factor to hazelnut production in the United States (Thompson et al., 1996). The disease is caused by the obligate biotrophic fungus *Anisogramma anomala*; an ascomycete in the order Diaporthales that is host specific to genus *Corylus* (Johnson and Pinkerton, 2002). *Anisogramma anomala*’s ascospores penetrate actively growing shoot tips during wet conditions in spring, and due to a long latent period, express no disease signs or symptoms for the first year of infection; 16-18 months later stem cankers develop (Gottwald and Cameron, 1979; Pinkerton et al., 1998a, b; Johnson and Pinkerton, 2002). Infections are characterized by sunken lesions lined with conspicuous gray to black colored, elliptical, 2-3 mm long stromata that erupt through the bark of shoots, causing branch dieback and potentially tree death (Pinkerton et al., 1993; Johnson and Pinkerton, 2002). The pathogen is native to the eastern United States where it persists in its natural host *Corylus americana*, the wild American hazelnut. While most plants of *C. americana* are highly tolerant to EFB, their other horticultural traits are lacking and do not fit traditional production systems (Fuller, 1908; Weschcke, 1954; Mehlenbacher, 1991; Capik and Molnar, 2012; Revord et al., 2020). The cultivated European hazelnut, *C. avellana*, is the species of global commerce and production cultivars yield large crops of marketable nuts, but unfortunately as a species *C. avellana* is largely very susceptible to EFB (Mehlenbacher and Olsen, 1997; Pinkerton et al., 1998a; Pinkerton et al., 1998b).

In the United States, ~99% of hazelnut production occurs in Oregon’s Willamette Valley (USDA NASS, 2024). This region is outside the native range of *A. anomala*, and for decades the historic hazelnut industry thrived EFB free in the valley’s Mediterranean-like climate. In the 1960s, however, *A. anomala* was inadvertently introduced to the Pacific Northwest (PNW) (Davison and Davidson, 1973). Whole orchards were decimated as the fungus spread southward towards Oregon from its point of introduction in southwest Washington state (Cameron, 1976; Gottwald and Cameron, 1980). EFB devastated most of the industry in Washington, especially since control measures were not available at the time. However, over the next decade, as EFB spread throughout the Willamette Valley, management approaches were developed that allowed some orchards to remain in production through heavy fungicide applications, scouting for cankers, and the removal, destruction, and burning of infected limbs (Johnson et al., 1996). These measures substantially increased production costs on an otherwise low-input crop (Julian et al., 2008), and thus, the development of cultivars resistant to EFB is considered the best approach to sustainably manage the disease (Thompson et al., 1996; Mehlenbacher and Molnar, 2021).

Fortunately, genetic resistance to EFB in the PNW was discovered in the obsolete European hazelnut pollinizer ‘Gasaway’ (Cameron, 1976). While ‘Gasaway’ itself produces small, oblong nuts, when it is crossed with a susceptible parent it transmits a dominant allele for EFB resistance to half of its offspring (Mehlenbacher et al., 1991). Breeding efforts at Oregon State University (OSU), Corvallis, Oregon, USA, successfully utilized ‘Gasaway’ as a parent in a modified backcross program, introgressing the single dominant resistance allele (R-gene) into commercial quality cultivars. ‘Gasaway’ R-gene carriers include the production cultivars Jefferson, Santiam, Yamhill, McDonald, PollyO, Dorris, and Wepster and the pollinizers Gamma, Delta, Zeta, Theta, Eta, Epsilon, York and Felix (Mehlenbacher and Smith, 2004; Mehlenbacher et al., 2007; Mehlenbacher et al, 2009; Mehlenbacher et al, 2011; Mehlenbacher et al, 2018). These resistant cultivars have been widely adopted and have revitalized the industry in Oregon, which has grown from 12,140 ha in 2009 to more than 40,000 ha today (Mehlenbacher, 2018; Mehlenbacher et al., 2023, N. Wiman, personal comm.).

Subsequent concerns over the durability of single gene resistance coupled with the desire to increase diversity in the breeding program has driven extensive germplasm curation and EFB screening efforts. Research at OSU has screened hundreds of *C. avellana* accessions from the germplasm collections of the breeding program and the USDA National Clonal Germplasm Repository, Corvallis, OR (Mehlenbacher, 2018). Foreign germplasm collection trips also yielded many seedlots for selection of superior seedlings and subsequent EFB screening at OSU, with many additional sources of EFB resistance identified in similar screening efforts at Rutgers University, New Brunswick, NJ (Molnar et al., 2007; Molnar and Pisetta, 2009; Capik et al., 2013; Leadbetter et al., 2015; Leadbetter et al., 2016; Molnar et al., 2018). This body of work has resulted in the identification of over 100 new accessions of diverse origin that are resistant or highly tolerant to EFB (Coyne et al., 1998; Lunde et al., 2000; Chen et al., 2007; Sathuvalli et al., 2010; Molnar et al., 2018; Mehlenbacher et al., 2023; Smith and Mehlenbacher, 2023).

Furthermore, genetic mapping studies have led to the identification of EFB R-gene loci on 3 of *C. avellana*’s 11 linkage groups (LGs). Notably, the ‘Gasaway’ resistance allele has been localized to LG6 (Mehlenbacher et al., 2006), along with resistant genotypes ‘Crvenje’ and ‘Uebov’ from Serbia (Colburn et al., 2015; Bhattarai et al., 2017a), ‘Culplà’ from Spain, OSU selections OSU 408.040 and OSU 533.129 from Minnesota and Michigan, respectively (Sathuvalli et al., 2012; Komaei Koma et al., 2021), several selections from the Russian federation and Crimea (Colburn et al., 2015; Komaei Koma et al., 2021; Mehlenbacher et al., 2023), and *Corylus heterophylla* ‘Ogyoo’ of Korea (Komaei Koma et al., 2021). Accessions from the Republic of Georgia, Holmkijj and Sochi, Russia, and Giresun, Turkey have been mapped to LG2 (Sathuvalli et al., 2011a; Sathuvalli et al., 2011b; Honig et al., 2019; Sekerli et al., 2021; Mehlenbacher et al., 2023). Additionally, Spanish cultivar ‘Ratoli’, *C. americana* ‘Rush’ and interspecific hybrid Yoder #5, and accessions from Moscow and Sochi, Russia, and Crimea have all had their R-genes mapped to LG7 (Sathuvalli et al., 2011a, b; Bhattarai et al., 2017b; Honig et al., 2019; Sekerli et al., 2021; Mehlenbacher et al., 2023).

The OSU breeding program has also identified multiple sources of quantitative resistance (QR), which manifests itself as reduced disease incidence, smaller cankers, and fewer numbers of cankers, when compared to susceptible cultivars (Coyne et al., 1998). Cultivars expressing QR include ‘Tonda di Giffoni’ and ‘Mortarella’ from Italy, ‘Sant Pere’ and ‘Closca Molla’ from Spain, and seedling selections from Turkey (n=17), Armenia (n=5), Azerbaijan (n=4), Southern Russia (n=5), the Republic of Georgia (n=4), and Crimea (n=1) (Pinkerton et al., 1993; Mehlenbacher et al., 2023). Notably, ‘Tonda di Giffoni’ has been shown to develop significantly lower levels of EFB than susceptible cultivars in New Jersey (Capik and Molnar, 2012). Lombardoni (2022) detected resistance QTL on LG10 of ‘Tonda di Giffoni’ in three different populations, by crossing the cultivar with three different *C. americana* selections. The percentage of phenotypic variation explained by the most important QTL on LG10 was 25.7% in the “CRA” population, followed by 18.9% and 16.2% in the “CRB” and “CRC” populations, respectively. ‘Sacajawea’, an offspring of ‘Sant Pere’, also expresses QR to EFB (Mehlenbacher et al., 2008) and is a parent of the recent releases ‘Monmouth’ and ‘Hunterdon’ that express very high levels of tolerance (Molnar, 2022).

Genetic diversity studies incorporating many of these resistant cultivars and selections support high genetic diversity among the material available for use in breeding programs, with resistance sources identified in nearly all of the distinct genetic clades resolved by Muehlbauer et al. (2014) and Lombardoni (2022). Overall, this wide diversity in EFB resistance sources is promising for resistance pyramiding and maintaining genetic diversity in future breeding efforts.

Of concern, however, is the potential presence of pathogenic variation in *A. anomala*, which can impact resistance screening and cultivar development efforts. In eastern North America, *A. anomala* exhibits high genetic diversity, whereas samples collected from the PNW were shown to be genetically homogenous (Muehlbauer et al., 2019; Tobia et al., 2024). Additionally, previous studies at Rutgers identified infections on ‘Gasaway’ and its offspring in greenhouse and field experiments, indicating possible pathogenic differences between isolates of *A. anomala* found in Oregon, New Jersey, and other eastern locations (Molnar et al., 2010a; Molnar et al., 2010b; Capik and Molnar, 2012). More recent field observations in New Jersey have shown significant EFB development on ‘Gasaway’-carrying cultivars (T. Molnar, personal communication), beyond that first reported in Capik and Molnar (2012), which indicates the presence of *A. anomala* populations able to overcome this R gene. Preliminary greenhouse inoculation experiments supported this premise, where *A. anomala* harvested from cankers on ‘Gasaway’-carrying trees was able to incite greater amounts of EFB and very large cankers on multiple ‘Gasaway’-carrying cultivars compared to *A. anomala* inoculum derived from non-R gene-containing host plants (Dunlevy et al., 2019).

These differences in resistance expression between Oregon and New Jersey, in the greenhouse and the field, support the presence of pathogenic variation and thus offer an opportunity for screening selections for EFB response under conditions that may support selection for more durable forms of resistance. The objective of this study is to document the EFB response of a large and diverse set of cultivars and selections deemed resistant or highly tolerant in Oregon when they are exposed to *A. anomala* in New Jersey. Results of the study will aid understanding of pathogenic variation and identity sources of resistance that maintain their status against New Jersey *A. anomala* populations in support of further breeding efforts and cultivar releases.

## Materials and method

### Plant materials

Trees were obtained in the winters of 2017 and 2019 as 1-year-old dormant bare root layers asexually propagated by tie-off layering (mound layering) at OSU. The 82 cultivars and selections included in this study (**Table 1**) were previously deemed resistant or highly tolerant to EFB at OSU following screening as part of their evaluations. Linkage group assignment of these resistance sources occurred across a large body of previous research, which is summarized by Mehlenbacher et al. (2023) and detailed in **Table 1**. We note that five selections are listed in **Table 1** as ‘unmapped’, reflecting instances where phenotypic evaluations in segregating progeny support major gene segregation patterns (often 1:1), but genetic mapping has yet to be performed to assign resistance to a linkage group. Layers were potted into 1 gal. (3.7 L) containers in peat-based growing media (Promix, Premier Tech Growers and Consumers, Quakertown, PA) and maintained in the greenhouse at 18 to 24 °C and 16-hour day lengths until June, when they were moved to a structure under 40% shade for acclimation. Trees were planted into a Sassafras sandy loam in October at the Rutgers University Horticultural Farm Three in East Brunswick, New Jersey. The farm is in USDA Hardiness Zone 7b and received an average of 128 mm of rain annually over the past five years. The 2017 planting consisted of 38 selections with four trees of each planted. The experiment contained a single block and one tree (clone) per genotype per replication, where complete randomization occurred with replication. Trees were spaced at 0.92 m in the row and 2.4 m between rows. The 2019 experiment consisted of 49 genotypes (11 cultivars and 38 selections) with 8-10 trees in 2 equal blocks of 4-5 trees per block, and statistical replications contained one tree per selection. Trees in this experiment were spaced 0.92 m within rows and 3 m between rows. Trees were assigned to positions randomly within each block. ‘Sacajawea’ and ‘Tonda di Giffoni’, both known to express EFB tolerance (QR) in Oregon and New Jersey, were included in each planting as controls. All trees were pruned to and maintained as a single stem, irrigated using orchard drip tubing, fertilized following standard practices of the Rutgers hazelnut breeding program, and provided with weed control as needed. No chemical controls for EFB or other pests or diseases were applied.

**Table 1:** Cultivars and selections evaluated for eastern filbert blight response in New Jersey.

Cultivar/Selection	Year of trial (No. Trees)^a^	Origin/Parentage^c^	Resistance Source^b^	LG^d^
OSU 1492.080	2017 (2); 2019 (10)	OSU 1085.073 x OSU 1039.064	Georgian, Geo 759.010	2 (Sathuvalli et al, 2011a; Sathuvalli et al, 2011b)
OSU 1502.111	2017 (4)	OSU 1119.081 x OSU 1038.084	Georgian, Geo 759.010	2
OSU 1440.026	2019 (10)	OSU 1085.066 x OSU 965.067	Georgian, Geo 759.010	2
OSU 1440.053	2019 (10)	OSU 1085.066 x OSU 965.067	Georgian, Geo 759.010	2
OSU 1456.062	2017 (4); 2019 (10)	OSU 1085.066 x OSU 1051.038	Georgian, Geo 759.010	2
OSU 1477.047	2017 (4); 2019 (10)	OSU 1085.066 x OSU 965.067	Georgian, Geo 759.010	2
OSU 1187.101	2019 (10)	Russia, Holmskij #2	Holmskij #2	2 (Sekerli et al., 2021)
OSU 1240.131	2017 (4); 2019 (10)	Turkey, Giresun 238	Turkish Giresun 238	2 (Mehlenbacher et al., 2023)
OSU 1289.028	2019 (10)	Turkey, Giresun 328	Turkish Giresun 328	2
OSU 1173.034	2019 (10)	OSU 747.104 x OSU 665.123	*C. heterophylla* ‘Ogyoo’	6 (Komaei Koma et al., 2021)
OSU 1495.017	2019 (8)	OSU 1181.023 x OSU 1029.039	*C. heterophylla* ‘Ogyoo’	6
OSU 1495.048	2019 (6)	OSU 1183.023 x OSU 1053.089	*C. heterophylla* ‘Ogyoo’	6
OSU 1495.063	2019 (8)	OSU 1183.023 x OSU 1053.089	*C. heterophylla* ‘Ogyoo’	6
OSU 1516.009	2017 (3)	OSU 1181.023 x OSU 1053.089	*C. heterophylla* ‘Ogyoo’	6
OSU 1185.126	2019 (8)	Crimea, AluSim #5	Crimea	6 (Komaei Koma et al., 2021)
Crvenje	2019 (9)	Serbia	Crvenje	6 (Colburn et al., 2015)
OSU 1350.055	2019 (10)	OSU 675.028 x Crvenje	Crvenje	6
OSU 1357.047	2019 (10)	OSU 713.068 x Crvenje	Crvenje	6
OSU 1358.058	2019 (10)	OSU 675.028 x Crvenje	Crvenje	6
OSU 1300.048	2019 (9)	OSU 612.015 x Culplà	Culpa	6 (Colburn et al., 2015)
OSU 1300.073	2019 (10)	OSU 612.015 x Culplà	Culpa	6
OSU 1352.059	2019 (10)	OSU 753.054 x Farris 533.029	Farris	6 (Komaei Koma et al., 2021)
OSU 1390.008	2019 (10)	OSU 753.054 x Farris 533.029	Farris	6
Dorris	2019 (9)	Delta x OSU 309.074	Gasaway	6 (Mehlenbacher et al., 2006)
Epsilon	2019 (10)	OSU 350.089 x Zimmerman	Gasaway	6
Felix	2019 (9)	OSU 384.095 X Delta	Gasaway	6
Gamma	2019 (10)	Casina x VR 6-28	Gasaway	6
Jefferson	2019 (10)	OSU 252.146 x OSU 414.062	Gasaway	6
McDonald	2019 (10)	Tonda Pacifica x Santiam	Gasaway	6
Yamhill	2019 (9)	OSU 296.082 x VR 8-32	Gasaway	6
Zeta	2019 (10)	OSU 342.019 x Zimmerman	Gasaway	6
OSU 1455.052	2019 (9)	OSU 1030.074 x OSU 962.014	OSU 408.040	6 (Sathuvalli et al., 2012)
OSU 1455.081	2017 (4)	OSU 1030.074 x OSU 962.014	OSU 408.040	6
OSU 1086.145	2019 (10)	OSU 713.068 x OSU 495.072	Russian 495.072	6 (Colburn et al., 2015)
OSU 1494.067	2017 (4)	OSU 1154.027 x OSU 1029.039	Russian 495.072	6
OSU 1509.111	2019 (10)	OSU 1154.027 x OSU 1029.039	Russian 495.072	6
OSU 1356.050	2019 (10)	OSU 679.114 x Uebov	Uebov	6 (Bhattarai et al., 2017a; Bhattarai et al., 2017b)
OSU 1399.091	2019 (9)	OSU 741.105 x Uebov	Uebov	6
Uebov	2019 (10)	Cacak, Serbia	Uebov	6
OSU 1086.053	2019 (10)	OSU 541.147 x OSU 665.123	*C. americana ‘Rush’*	7 (Bhattarai et al., 2017a; Bhattarai et al., 2017b)
OSU 1496.008	2019 (10)	OSU 1086.053 x OSU 1053.089	*C. americana ‘Rush’*	7
OSU 1505.116	2017 (4)	OSU 1086.053 x OSU 1053.089	*C. americana ‘Rush’*	7
OSU 541.147 “The Beast”	2019 (10)	NY 616 x OSU 226.118	*C. americana ‘Rush’*	7
OSU 1062.055	2019 (10)	Yoder#5 x OSU 616.018	Hybrid Yoder#5	7 (Bhattarai et al., 2017a; Bhattarai et al., 2017b)
OSU 1457.074	2017 (4)	OSU 1062.055 x OSU 978.058	Hybrid Yoder#5	7
OSU 1026.073	2019 (10)	OSU 665.123 x Ratoli	Ratoli	7 (Sathuvalli et al., 2011a; Bhattarai et al., 2017b)
OSU 1382.092	2019 (10)	OSU 1011.001 x OSU 937.069	Ratoli	7
OSU 1413.032	2019 (10)	OSU 1011.001 x OSU 963.077	Ratoli	7
OSU 1443.080	2019 (9)	OSU 1026.073 x OSU 1039.064	Ratoli	7
OSU 1443.083	2017 (4)	OSU 1026.073 x OSU 1039.064	Ratoli	7
OSU 1166.123	2019 (10)	Russia, Sochi Institute Redleaf	Sochi Redleaf	7 (Sekerli et al., 2021)
OSU 889.084	2019 (10)	OSU 401.014 x Tonda Pacifica	Bauman Hybrid 401.014	Unmapped
OSU 1343.034	2019 (10)	OSU 539.031 x CTG11	Gellatly Tree Hazel #11 (CTG11)	Unmapped
OSU 1168.013	2019 (10)	Russia, Holmskij #4	Holmskij #4	Unmapped
OSU 1231.091	2019 (10)	Turkey, Giresun 230	Turkish Giresun 230	Unmapped
OSU 1233.145	2017 (4); 2019 (10)	Turkey, Giresun 530	Turkish Giresun 520	Unmapped
OSU 1012.074	2017 (4)	OSU 556.166 x Closca Molla	Quantitative Resistance	–
OSU 1030.092	2017 (4)	OSU 693.117 x OSU 612.015	Quantitative Resistance	–
OSU 1092.032	2017 (4)	Azerbaijan, Qabala Market	Quantitative Resistance	–
OSU 1096.067	2017 (2)	OSU 444.027 x OSU 664.147	Quantitative Resistance	–
OSU 1120.020	2017 (4)	Republic of Georgia, Kakheti	Quantitative Resistance	–
OSU 1122.018	2017 (4)	Azerbajan, Ata Baba #2	Quantitative Resistance	–
OSU 1166.108	2017 (4)	Russia, Holmskij #2	Quantitative Resistance	–
OSU 1171.027	2017 (3)	Armenia, ARM 173	Quantitative Resistance	–
OSU 1186.123	2017 (4)	Armenia, ARM 051	Quantitative Resistance	–
OSU 1225.046	2017 (4)	Republic of Georgia, Abasha	Quantitative Resistance	–
OSU 1229.082	2017 (4)	Turkey, Giresun 183	Quantitative Resistance	–
OSU 1232.138	2017 (2)	Turkey, Giresun 350	Quantitative Resistance	–
OSU 1459.054	2017 (4)	OSU 786.091 x OSU 961.070	Quantitative Resistance	–
OSU 1460.006	2017 (4)	OSU 786.091 x OSU 1012.074	Quantitative Resistance	–
OSU 693.040	2017 (4)	Turkey, Akçakoca	Quantitative Resistance	–
OSU 693.121	2017 (4)	Turkey, Yomra	Quantitative Resistance	–
OSU 702.004	2017 (4)	Turkey, Yomra	Quantitative Resistance	–
OSU 702.031	2017 (4)	Turkey, Yomra	Quantitative Resistance	–
OSU 702.041	2017 (2)	Turkey, Yomra	Quantitative Resistance	–
OSU 717.087	2017 (4)	Turkey, Akçakoca	Quantitative Resistance	–
OSU 723.042	2017 (4)	Turkey, Yomra	Quantitative Resistance	–
OSU 786.091	2017 (4)	OSU 256.005 x OSU 439.063	Quantitative Resistance	–
OSU 938.054	2017 (4)	OSU 539.031 x OSU 474.013	Quantitative Resistance	–
OSU 978.064	2017 (4)	OSU 556.019 x Sacajawea	Quantitative Resistance	–
Sacajawea	2017 (3); 2019 (8)	OSU 43.091 x Sant Pere	Quantitative Resistance	–
Tonda di Giffoni	2017 (4)	Italy	Quantitative Resistance	–

a Year of trial establishment and number of replications per trial. Note some cultivars/selections are repeated across both years.

b Suspected source of eastern filbert blight resistance, including R-gene donor or quantitatively inherited.

c Location of origin for foreign introductions or pedigree for breeding selections.

d Linkage group assignment of R-gene, and reference to the study in which genetic mapping was performed. ‘Unmapped’ applies to suspected R-gene carriers based upon observed progeny segregation patterns that fit a 1:1 pattern but where linkage group assignment has not yet been performed. “-”for cultivars/selections expressing quantitative resistance.

### Exposure to EFB

Trees were exposed to *A. anomala* via field inoculations as detailed in Molnar et al. (2007). In the early winter, stems containing cankers with intact stromata were collected from EFB infected trees located on Rutgers’ Horticulture Research Farms. Cankers were cut into ~15 cm sections and stored in doubled polyethylene bags at -6 °C until the early spring, at which point the stems were tied into the canopy of each tree. Trees were also exposed to EFB via natural spread from susceptible trees in adjacent plots and then from individuals that became infected within the trials. Tobia et al. (2024) documented the high diversity of local *A. anomala*.

### EFB evaluations and measurements

Two years after planting and for the following three years, trees were visually inspected for EFB disease incidence. Each individual tree was given a disease rating from 0-5, as developed by Pinkerton et al. (1992), by which a score of 0= no EFB present, 1 = 1 canker, 2= multiple cankers on the same limb, 3= multiple limbs with cankers, 4= majority of limbs expressing EFB, and 5= all limbs have cankers and/or presence of dead limbs. Incidence of canopy death and tree fatality were also noted.

On each tree expressing EFB, the total number of cankers (NC) were counted. Each canker was measured; total canker length (TCL) was the sum of individual measurements. TCL was divided by NC to calculate mean canker length (MCL). Total shoot length (TSL) (excluding the current season’s growth, which does not express EFB due to *A. anomala’*s latency) was also measured. TCL was square root transformed (Square root total canker length; SRCL) to equalize variance and improve normalization for mean comparisons, as with previous data of this type (Coyne et al., 2000; Lombardoni, 2022; Revord et al., 2020). After transformation, TCL was divided by TSL to calculate the proportion of each tree that is covered in cankers (proportion of diseased wood; PDW) (Osterbauer et al., 1997; Capik and Molnar, 2012). Mean TCL, mean TSL, and mean PDW were calculated for each cultivar/selection using only their respective trees that expressed measurable EFB, while mean EFB rating (0-5) was calculated inclusive of all trees of a given cultivar/selection.

One-way analysis of variance (ANOVA) was performed, respectively, for each experimental trial on EFB rating, NC, MCL, SRCL, and PDW (i.e., 2017 and 2019) using the ‘agricolae’ package in R-studio and a significance threshold of p≤.05 (de Mendiburu and Yaseem, 2019; Rstudio Team, 2022). Tukey’s Honestly Significant Difference test was performed *post hoc* to assess for pairwise differences among cultivars and selections, within respective trials, for all phenotypes.

## Results

**Figure 1:**
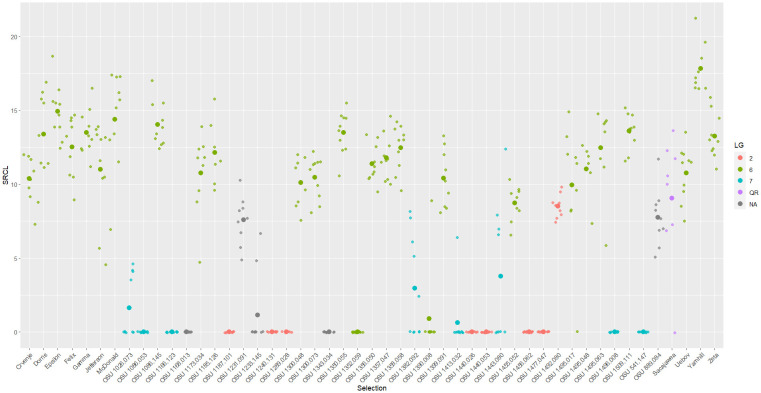
Scatter plot of square root total canker length (SRCL) measured on cultivars and selections in the 2019 replicated trial. Heavier dots signify cultivar/selection means while lighter dots signify values of individual trees. Colors denote linkage group or resistance source.

**Figure 2:**
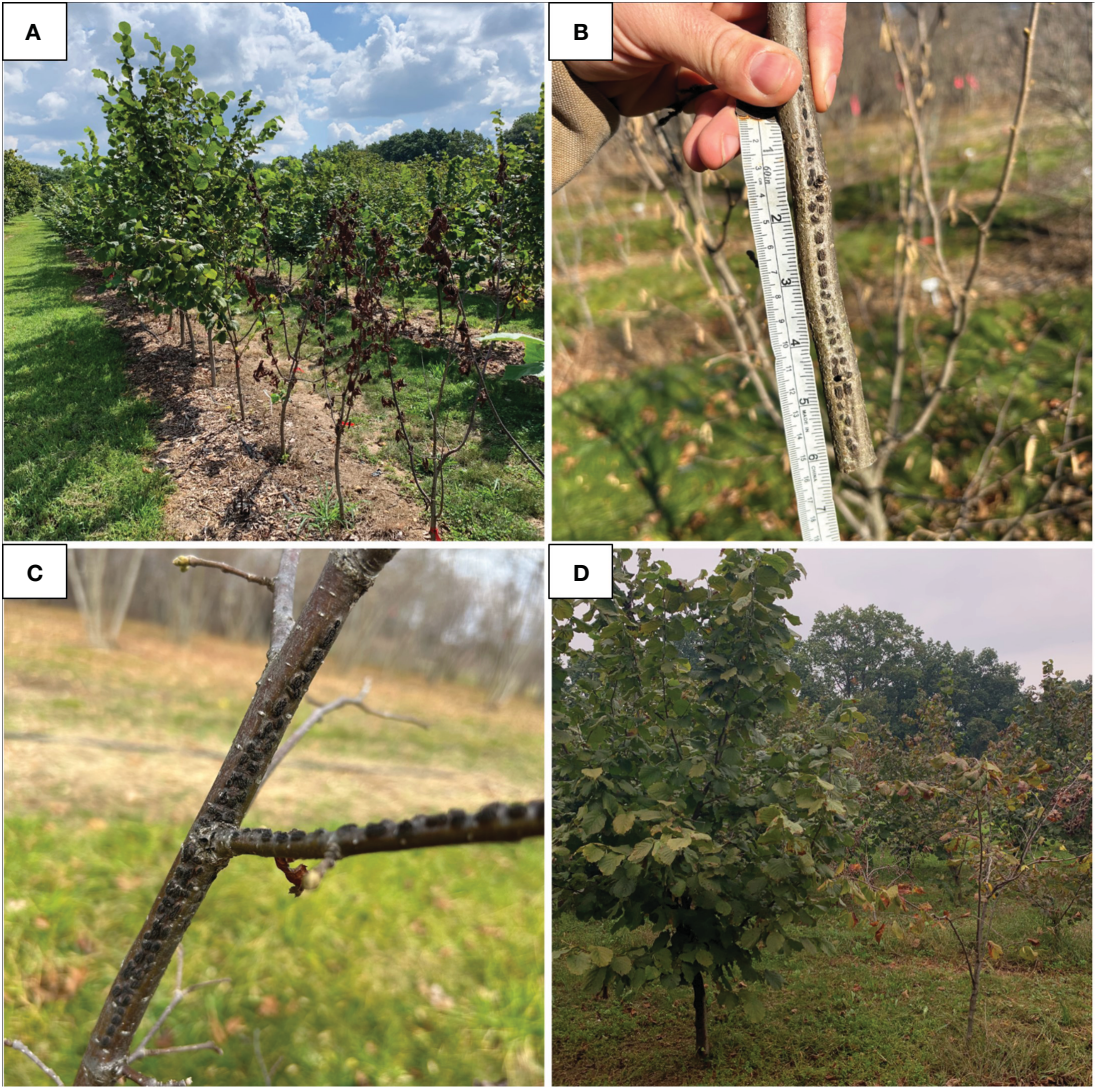
Images of eastern filbert blight expression in the 2017 and 2019 trials. **(A)** Eastern filbert blight expression of trees with overcome R-genes (foreground) next to health trees (background) in the 2019 trial. **(B)** A typical eastern filbert blight canker typical found on ‘Ratoli’ offspring in this study. **(C)** An eastern filbert blight canker found on ‘Ratoli’ offspring OSU 1443.080. **(D)** Variation of eastern filbert blight expression in quantitatively resistant selections in the 2017 trial with a tree of OSU 1460.006 exhibiting high tolerance (left) and a tree of OSU 723.042 exhibiting moderately low tolerance (right).

Overall, EFB infections were widespread and uniform across the two plantings, with severely infected selections and cultivars present in both trials. Disease expression was generally consistent among trees of the same selection (**Tables 2** , **3**; **Figures 1**, **2**), indicating unlikelihood that any selections escaped exposure to EFB. The controls ‘Tonda di Giffoni’ and ‘Sacajawea’ developed mild and moderate EFB symptoms, respectfully, with all ‘Tonda di Giffoni’ trees and all ‘Sacajawea’ trees across the trials developing disease (**Figure 1**). The PDW of ‘Tonda di Giffoni’ was 0.053 in the 2017 trial (n=4). ‘Sacajawea’ developed more severe EFB than ‘Tonda di Giffoni,’ with an average PDW of 0.215 across all plantings (n=12). In total, 75% (108 of 144) and 60% (287 of 480) of trees expressed EFB in the 2017 and 2019 trials, respectively. The mean EFB rating of infected trees in the 2017 experiment was 3.8 out of 5.0 and mean PDW was 0.227. Similarly, the 2019 experiment had a mean score of 4.0 and a PDW of 0.334. These findings demonstrate significant variation in the disease response of some of the cultivars/selections between Oregon, where they developed little or no EFB, and their response in New Jersey, where many succumbed to EFB.

**Table 2:** Eastern filbert blight response of cultivars and selections planted in the 2017 replicated trial.

Cultivar/Selection	EFB Rating^ag^	No. of Cankers^bg^	Mean canker length (cm)^cg^	Square Root Total Canker Length (√cm)^dg^	Proportion of Diseased Wood^eg^	Resistance Source^f^
OSU 1456.062	0.0^a^ ± 0.0	0.0^a^ ± 0.0	0.0^a^ ± 0.0	0.0^a^ ± 0.0	0.000^a^ ± 0.000	LG2 Geo. 759.010
OSU 1477.047	0.0^a^ ± 0.0	0.0^a^ ± 0.0	0.0^a^ ± 0.0	0.0^a^ ± 0.0	0.000^a^ ± 0.000	LG2 Geo. 759.010
OSU 1502.111	0.0^a^ ± 0.0	0.0^a^ ± 0.0	0.0^a^ ± 0.0	0.0^a^ ± 0.0	0.000^a^ ± 0.000	LG2 Geo. 759.010
OSU 1240.131	0.0^a^ ± 0.0	0.0^a^ ± 0.0	0.0^a^ ± 0.0	0.0^a^ ± 0.0	0.000^a^ ± 0.000	LG2 Giresun 238
OSU 1505.166	0.0^a^ ± 0.0	0.0^a^ ± 0.0	0.0^a^ ± 0.0	0.0^a^ ± 0.0	0.000^a^ ± 0.000	LG7 *C. americana* ‘Rush’
OSU 1457.074	0.0^a^ ± 0.0	0.0^a^ ± 0.0	0.0^a^ ± 0.0	0.0^a^ ± 0.0	0.000^a^ ± 0.000	LG7 ‘Yoder #5’
OSU 1443.083	0.0^a^ ± 0.0	0.0^a^ ± 0.0	0.0^a^ ± 0.0	0.0^a^ ± 0.0	0.000^a^ ± 0.000	LG7 Ratoli
OSU 1494.067	5.0^g^ ± 0.0	8.5^dg^ ± 3.5	13.1^ad^ ± 2.4	10.3^ch^ ± 1.3	0.150^ag^ ± 0.000	LG6 Russian 495.072
OSU 1233.145	2.0^cd^ ± 0.8	2.8^ad^ ± 1.5	15.0^ad^ ± 2.6	6.0^ad^ ± 1.5	0.023^ab^ ± 0.015	NA Giresun 530
OSU 1492.080	5.0^g^ ± 0.0	7.0^cg^ ± 1.4	21.0^ad^ ± 16.3	11.8^ci^ ± 6.1	0.390^gh^ ± 0.297	LG2 Geo. 759.010
OSU 1455.081	4.5^g^ ± 1.0	6.3^bg^ ± 5.4	33.1^de^ ± 15.7	13.0^ci^ ± 5.3	0.343^fh^ ± 0.232	LG6 OSU 408.040
OSU 1516.009	4.8^g^ ± 0.5	10.0^fg^ ± 1.7	37.1^de^ ± 15.8	18.8^ij^ ± 3.0	0.507^hi^ ± 0.119	LG6 *C. heterophylla* Ogyoo
OSU 1460.006	0.3^ab^ ± 0.5	0.3^a^ ± 0.5	1.7^ab^ ± 3.5	0.6^ab^ ± 1.3	0.003^a^ ± 0.005	QR
OSU 1186.123	0.3^ab^ ± 0.5	0.3^a^ ± 0.5	2.5^ac^ ± 5.0	0.8^ab^ ± 1.6	0.003^a^ ± 0.127	QR
OSU 1229.082	2.0^cd^ ± 1.8	1.3^ab^ ± 1.0	22.2^ad^ ± 26.5	5.1^ac^ ± 4.6	0.090^af^ ± 0.098	QR
OSU 1122.018	1.8^bc^ ± 1.5	1.3^ab^ ± 1.0	23.6^ae^ ± 16.0	5.4^ad^ ± 3.7	0.063^ae^ ± 0.049	QR
OSU 938.054	2.0^cd^ ± 0.8	2.5^ac^ ± 1.0	13.1^ad^ ± 5.7	5.6^ad^ ± 2.1	0.025^ab^ ± 0.013	QR
OSU 1012.074	2.3^de^ ± 1.0	1.8^ab^ ± 0.5	29.2^ae^ ± 13.2	7.1^be^ ± 2.3	0.048^ac^ ± 0.015	QR
Tonda di Giffoni	3.0^df^ ± 0.0	3.5^ae^ ± 0.6	16.5^ad^ ± 6.3	7.6^cf^ ± 2.0	0.053^ad^ ± 0.028	QR
OSU 723.042	4.5^g^ ± 0.6	5.0^af^ ± 1.8	19.0^ad^ ± 6.8	9.3^cg^ ± 1.1	0.143^ag^ ± 0.034	QR
OSU 693.040	5.0^g^ ± 0.0	6.3^bg^ ± 1.5	19.3^ad^ ± 9.2	10.7^ch^ ± 2.1	0.240^ah^ ± 0.044	QR
OSU 978.064	3.5^dg^ ± 1.3	4.5^af^ ± 2.4	29.2^ae^ ± 4.6	11.0^ch^ ± 2.4	0.203^ag^ ± 0.153	QR
OSU 702.004	3.8^eg^ ± 1.0	4.0^af^ ± 1.4	33.6^de^ ± 12.8	11.3^ch^ ± 2.5	0.160^ag^ ± 0.067	QR
OSU 1232.138	4.8^g^ ± 0.5	3.5^ae^ ± 0.7	37.5^de^ ± 9.5	11.3^ci^ ± 0.3	0.225^ah^ ± 0.092	QR
OSU 1030.092	4.3^fg^ ± 0.5	5.3^af^ ± 2.8	28.0^ae^ ± 8.1	11.5^ci^ ± 3.7	0.298^ch^ ± 0.164	QR
OSU 693.121	4.5^g^ ± 0.6	4.5^af^ ± 1.3	31.3^ce^ ± 2.9	11.8^ci^ ± 2.0	0.218^ag^ ± 0.075	QR
OSU 702.041	5.0^g^ ± 0.0	6.0^ag^ ± 1.4	28.7^ae^ ± 4.1	13.2^di^ ± 2.5	0.355^fh^ ± 0.120	QR
OSU 702.031	5.0^g^ ± 0.0	7.0^cg^ ± 3.6	30.4^be^ ± 17.0	13.3^ei^ ± 1.4	0.258^ah^ ± 0.067	QR
OSU 1092.032	3.5^dg^ ± 0.6	5.0^af^ ± 0.8	37.2^de^ ± 6.7	13.5^ej^ ± 1.4	0.155^ag^ ± 0.041	QR
OSU 1166.108	5.0^g^ ± 0.0	3.8^af^ ± 0.5	52.9^e^ ± 12.8	14.0^fj^ ± 2.3	0.320^eh^ ± 0.130	QR
OSU 786.091	5.0^g^ ± 0.0	8.0^cg^ ± 4.7	35.4^de^ ± 20.1	14.6^gj^ ± 2.7	0.273^bh^ ± 0.075	QR
OSU 717.087	4.8^g^ ± 0.5	7.8^cg^ ± 2.5	30.9^be^ ± 7.2	15.2^gj^ ± 2.2	0.373^gh^ ± 0.100	QR
Sacajawea	4.8^g^ ± 0.5	9.7^eg^ ± 5.5	31.0^be^ ± 18.6	15.2^gj^ ± 0.9	0.167^ag^ ± 0.021	QR
OSU 1459.054	5.0^g^ ± 0.0	8.5^dg^ ± 2.4	32.7^de^ ± 7.7	16.5^hj^ ± 3.2	0.350^fh^ ± 0.185	QR
OSU 1225.046	5.0^g^ ± 0.0	11.3^g^ ± 1.7	24.6^ae^ ± 4.0	16.5^hj^ ± 1.2	0.310^dh^ ± 0.050	QR
OSU 1171.027	5.0^g^ ± 0.0	8.3^cg^ ± 3.1	40.7^de^ ± 11.4	17.8^hj^ ± 2.3	0.383^gh^ ± 0.127	QR
OSU 1096.067	5.0^g^ ± 0.0	13.0^g^ ± 1.4	27.6^ae^ ± 6.4	18.8^ij^ ± 1.1	0.775i ± 0.205	QR
OSU 1120.020	5.0^g^ ± 0.0	12.0^g^ ± 2.5	35.2^de^ ± 10.7	20.1j ± 1.1	0.535^hi^ ± 0.106	QR

a Eastern filbert blight rating on 0-5 scale as developed by Pinkerton et al. (1992).

b Mean number of EFB cankers per replicate (tree).

c Average length of individual EFB cankers per selection.

d Square root transformed mean total length of cankers across all replicates per selection.

e Proportion of shoots that are covered in EFB cankers, calculated as TCL/TSL.

f Suspected source of eastern filbert blight resistance with R-gene linkage group if known. Accessions with exhibiting quantitative resistance noted as “QR”.

g For the given attribute, means followed by a different letter in the same column are significantly different (P<.05) according to Tukey’s HSD.

**Table 3:** Eastern filbert blight response of cultivars and selections planted in the 2019 replicated trial.

Cultivar/Selection	EFB Rating^ag^	No. of Cankers^bg^	Mean Canker Length (cm)^cg^	Square Root Total Canker Length(√cm)^dg^	Proportion of Diseased Wood^eg^	Resistance source^f^
OSU 1240.131	0.0^a^ ± 0.0	0.0^a^ ± 0.0	0.0^a^ ± 0.0	0.0^a^ ± 0.0	0.000^a^ ± 0.000	LG2 Giresun 238
OSU 1289.028	0.0^a^ ± 0.0	0.0^a^ ± 0.0	0.0^a^ ± 0.0	0.0^a^ ± 0.0	0.000^a^ ± 0.000	LG2 Giresun 328
OSU 1187.101	0.0^a^ ± 0.0	0.0^a^ ± 0.0	0.0^a^ ± 0.0	0.0^a^ ± 0.0	0.000^a^ ± 0.000	LG2 Holmskij #2
OSU 1440.026	0.0^a^ ± 0.0	0.0^a^ ± 0.0	0.0^a^ ± 0.0	0.0^a^ ± 0.0	0.000^a^ ± 0.000	LG2 Geo. 759.010
OSU 1440.053	0.0^a^ ± 0.0	0.0^a^ ± 0.0	0.0^a^ ± 0.0	0.0^a^ ± 0.0	0.000^a^ ± 0.000	LG2 Geo. 759.010
OSU 1456.062	0.0^a^ ± 0.0	0.0^a^ ± 0.0	0.0^a^ ± 0.0	0.0^a^ ± 0.0	0.000^a^ ± 0.000	LG2 Geo. 759.010
OSU 1477.047	0.0^a^ ± 0.0	0.0^a^ ± 0.0	0.0^a^ ± 0.0	0.0^a^ ± 0.0	0.000^a^ ± 0.000	LG2 Geo. 759.010
OSU 1492.080	4.5^de^ ± 0.7	3.7^bg^ ± 0.9	20.8^df^ ± 6.6	8.5^ce^ ± 0.8	0.322^dg^ ± 0.081	LG2 Geo. 759.010
OSU 1352.059	0.0^a^ ± 0.0	0.0^a^ ± 0.0	0.0^a^ ± 0.0	0.0^a^ ± 0.0	0.000^a^ ± 0.000	LG6 Farris 533.029
OSU 1390.008	0.2^a^ ± 0.6	0.2^a^ ± 0.6	4.0^a^ ± 12.5	0.9^ab^ ± 2.8	0.009^a^ ± 0.027	LG6 Farris 533.029
OSU 1185.126	4.6^de^ ± 0.8	7.3^im^ ± 3.3	22.9^ef^ ± 7.4	12.1^ej^ ± 2.2	0.301^cg^ ± 0.114	LG6 AluSim#5
OSU 1173.034	4.1^de^ ± 1.1	6.6^gm^ ± 2.9	18.7^bd^ ± 5.4	10.8^ci^ ± 2.7	0.327^dh^ ± 0.170	LG6 *C. heterophylla* Ogyoo
OSU 1495.017	4.1^de^ ± 1.6	5.2^dj^ ± 2.9	20.6^df^ ± 9.9	9.9^cg^ ± 4.3	0.379^ei^ ± 0.176	LG6 *C. heterophylla* Ogyoo
OSU 1495.063	4.8^e^ ± 0.7	5.9^ek^ ± 2.7	29.6^ef^ ± 6.7	12.5^fj^ ± 2.9	0.384^ei^ ± 0.092	LG6 *C. heterophylla* Ogyoo
OSU 1495.048	5.0^e^ ± 0.0	5.1^dj^ ± 2.1	26.0^ef^ ± 7.1	11.0^cj^ ± 1.9	0.373^ei^ ± 0.100	LG6 *C. heterophylla* Ogyoo
Crvenje	4.1^ce^ ± 0.8	4.9^dj^ ± 1.3	23.4^ef^ ± 7.4	10.4^ch^ ± 1.5	0.232^be^ ± 0.095	LG6 Crvenje
OSU 1357.047	4.8^e^ ± 0.4	6.4^fk^ ± 1.9	24.7^ef^ ± 12.7	11.8^ej^ ± 1.5	0.402^fi^ ± 0.150	LG6 Crvenje
OSU 1358.058	4.8^e^ ± 0.4	7.7^im^ ± 3.1	23.1^ef^ ± 8.6	12.5^fj^ ± 1.5	0.329^eh^ ± 0.086	LG6 Crvenje
OSU 1350.055	5.0^e^ ± 0.0	6.5^gl^ ± 1.8	29.5^ef^ ± 6.5	13.5^hj^ ± 1.4	0.528^hj^ ± 0.182	LG6 Crvenje
OSU 1300.048	4.6^e^ ± 0.5	5.1^dj^ ± 1.7	20.5^cf^ ± 2.5	10.1^ch^ ± 1.7	0.327^dh^ ± 0.161	LG6 Culplà
OSU 1300.073	4.8^e^ ± 0.4	5.0^dj^ ± 1.5	23.5^ef^ ± 7.2	10.5^ch^ ± 1.4	0.383^ei^ ± 0.155	LG6 Culplà
Jefferson	4.2^de^ ± 1.7	4.9^dj^ ± 2.4	27.2^ef^ ± 5.4	11.0^cj^ ± 3.3	0.538^ij^ ± .215	LG6 Gasaway
McDonald	4.6^e^ ± 1.3	9.2^km^ ± 3.7	26.7^ef^ ± 10.5	14.4^jk^ ± 3.3	0.68^1j^ ± 0.290	LG6 Gasaway
Felix	4.7^e^ ± 0.5	8.2^km^ ± 2.8	19.0^ce^ ± 1.8	12.5^fj^ ± 2.1	0.289^cf^ ± 0.077	LG6 Gasaway
Gamma	4.8^e^ ± 0.4	9.3^lm^ ± 3.2	20.7^df^ ± 4.5	13.5^hj^ ± 1.6	0.344^ei^ ± 0.111	LG6 Gasaway
Zeta	5.0^e^ ± 0.0	7.1^hm^ ± 1.4	25.6^ef^ ± 5.7	13.3^gj^ ± 1.5	0.419^fi^ ± 0.080	LG6 Gasaway
Yamhill	5.0^e^ ± 0.0	13.7n ± 2.5	23.6^ef^ ± 2.8	17.8k ± 1.6	0.465^fi^ ± 0.106	LG6 Gasaway
Dorris	5.0^e^ ± 0.0	6.9^hm^ ± 2.8	27.2^ef^ ± 2.7	13.4^hj^ ± 2.8	0.511^hj^ ± .197	LG6 Gasaway
Epsilon	5.0^e^ ± 0.0	9.9m ± 3.3	24.5^ef^ ± 7.4	14.9k ± 1.8	0.385^ei^ ± 0.104	LG6 Gasaway
OSU 1455.052	4.8^e^± 0.7	2.6^ae^ ± 0.9	32.9^f^ ± 11.1	8.7^ce^ ± 1.2	0.372^ei^ ± 0.137	LG6 OSU 408.040
OSU 1509.111	4.7^e^ ± 0.5	8.1^km^ ± 1.2	23.1^ef^ ± 2.8	13.6^hj^ ± 1.2	0.399^fi^ ± 0.107	LG6 Russian 495.072
OSU 1086.145	5.0^e^ ± 0.0	8.0^jm^ ± 2.3	26.2^ef^ ± 7.0	14.0^ij^ ± 1.5	0.432^fi^ ± 0.191	LG6 Russian 495.072
Uebov	4.6^e^ ± 1.0	4.4^ch^ ± 1.5	27.4^ef^ ± 5.1	10.8^ci^ ± 1.8	0.389^ei^ ± 0.091	LG6 Uebov
OSU 1356.050	5.0^e^ ± 0.0	4.8^di^ ± 1.0	28.0^ef^ ± 6.9	11.4^dj^ ± 1.3	0.382^ei^ ± 0.128	LG6 Uebov
OSU 1399.091	5.0^e^ ± 0.0	5.1^dj^ ± 1.5	21.6^df^ ± 4.4	10.4^ch^ ± 2.0	0.500^gi^ ± 0.191	LG6 Uebov
OSU 1086.053	0.0^a^ ± 0.0	0.0^a^ ± 0.0	0.0^a^ ± 0.0	0.0^a^ ± 0.0	0.000^a^ ± 0.000	LG7 *C. americana* Rush
OSU 1496.008	0.0^a^ ± 0.0	0.0^a^ ± 0.0	0.0^a^ ± 0.0	0.0^a^ ± 0.0	0.000^a^ ± 0.000	LG7 *C. americana* Rush
OSU 541.147	0.0^a^ ± 0.0	0.0^a^ ± 0.0	0.0^a^ ± 0.0	0.0^a^ ± 0.0	0.000^a^ ± 0.000	LG7 *C. americana* Rush
OSU 1166.123	0.0^a^ ± 0.0	0.0^a^ ± 0.0	0.0^a^ ± 0.0	0.0^a^ ± 0.0	0.000^a^ ± 0.000	LG7 Sochi Redleaf
OSU 1413.032	0.1^a^ ± 0.3	0.1^a^ ± 0.3	4.1^a^ ± 13.0	0.6^ab^ ± 2.0	0.007^a^ ± 0.021	LG7 Ratoli
OSU 1026.073	0.4^a^ ± 0.5	0.4^a^ ± 0.5	6.8^ab^ ± 9.0	1.6^ab^ ± 2.1	0.010^a^ ± 0.013	LG7 Ratoli
OSU 1382.092	1.0^ab^ ± 1.2	1.2^ab^ ± 1.5	8.8^ac^ ± 12.4	3.0^ab^ ± 3.5	0.016^a^ ± 0.021	LG7 Ratoli
OSU 1443.080	1.1^ac^ ± 1.4	1.8^ac^ ± 2.5	8.4^ac^ ± 10.2	3.8^b^ ± 4.8	0.053^ab^ ± 0.073	LG7 Ratoli
OSU 1168.013	0.0^a^ ± 0.0	0.0^a^ ± 0.0	0.0^a^ ± 0.0	0.0^a^ ± 0.0	0.000^a^ ± 0.000	NA Holmskij #4
OSU 1343.034	0.0^a^ ± 0.0	0.0^a^ ± 0.0	0.0^a^ ± 0.0	0.0^a^ ± 0.0	0.000^a^ ± 0.000	NA Gellatly Tree Hazel #11
OSU 1233.145	0.2^a^ ± 0.4	0.2^a^ ± 0.4	6.8^ab^ ± 15.2	1.2^ab^ ± 2.5	0.011^a^ ± 0.024	NA Giresun 530
OSU 889.084	4.8^e^ ± 0.3	3.2^af^ ± 1.9	22.6^df^ ± 10.1	7.8^cd^ ± 1.9	0.431^fi^ ± 0.164	NA Bauman 401.014
OSU 1231.091	2.0^ac^ ± 1.0	2.2^ad^ ± 1.5	32.2^ef^ ± 13.6	7.6^c^ ± 1.6	0.118^ac^ ± 0.076	NA Giresun 230
Sacajawea	3.1^bd^ ± 1.7	4.0^bh^ ± 2.6	22.0^df^ ± 9.6	9.1^cf^ ± 4.3	0.177^ad^ ± 0.107	QR

a Eastern filbert blight rating on 0-5 scale as developed by Pinkerton et al. (1992).

b Mean number of EFB cankers per replicate (tree).

c Average length of individual EFB cankers per selection.

d Square root transformed mean total length of cankers across all replicates per selection.

e Proportion of shoots that are covered in EFB cankers, calculated as TCL/TSL.

f Suspected source of eastern filbert blight resistance with R-gene linkage group if known. Accessions with exhibiting quantitative resistance noted as “QR”.

g For the given attribute, means followed by a different letter in the same column are significantly different (P<.05) according to Tukey’s HSD.

### Major gene carrying cultivars and selections

#### Resistance genes mapped to LG6

The 2019 trial contained eight OSU cultivars (n= 8-10 trees each) that carry the ‘Gasaway’ R-gene, including ‘Jefferson’, ‘Yamhill’, ‘McDonald’, ‘Dorris’, ‘Felix’, ‘Epsilon’, ‘Gamma’, and ‘Zeta’. At the conclusion of the 2019 study in March 2023, every tree exhibited EFB and most had severe disease. In total, 77 ‘Gasaway’ R-gene carrying individuals exhibited an average PDW of 0.449, and 75 of 77 exhibited severe canopy dieback and/or total tree death due to EFB. Most cankers found on cultivars with ‘Gasaway’ resistance were typical, contained regular stromata, and expanded in length over the course of the study, which contrasts with findings in Oregon at the time that these plant materials were selected, where cankers of ‘Jefferson’ were non-typical and appeared to heal as the trees matured (Pscheidt et al., 2013; Pscheidt et al, 2022).

Trees of ‘McDonald’ developed the most severe disease of any cultivar or selection in the trial with a mean PDW of 0.681, and all trees of ‘Dorris’, ‘Jefferson’, and ‘McDonald’ had died from EFB by the conclusion of the trial (within 4 years of planting). Importantly, clones of ‘Dorris’, ‘McDonald’, ‘Jefferson’, ‘Yamhill’ and ‘Zeta’ all expressed mean PDW significantly greater (p-values<.05) than ‘Sacajawea’, whose 8 trees in the 2019 trial expressed a mean PDW of 0.177. ‘Felix’ and ‘Gamma’ had comparatively lower PDW among the cultivars with ‘Gasaway’ resistance, at 0.289 and 0.344, respectively, but the ‘Felix’ trees also died by the conclusion of the study. Interestingly, trees of ‘Gamma’ and ‘Epsilon’ exhibited some vigor despite severe EFB infections, where canopy dieback occurred, but disease was not fatal during the study period.

Eight additional LG6 sources of resistance that are unrelated to ‘Gasway’ were also tested, represented by 2 cultivars and 20 selections (**Table 1**). All but two selections developed severe or fatal disease (**Table 3**; **Figure 2A**). These selections, OSU 1352.059 and OSU 1390.008, both descend from OSU 533.029, which originates from amateur breeder Cecil Farris of Michigan (Farris, 1988; Farris, 1990). All trees of OSU 1352.059 grew vigorously and expressed no fruiting EFB cankers for the duration of the trial (**Table 3**). Only one tree of OSU 1390.008 developed sporulating cankers. The two cankers on the single tree totaled 40 cm in length and caused no related branch die-back (**Table 3**). Both selections, however, had three individual trees that exhibited sunken, atypical EFB lesions, although they were small (<30 cm) and did not appear to impact overall tree health.

**Figure 3:**
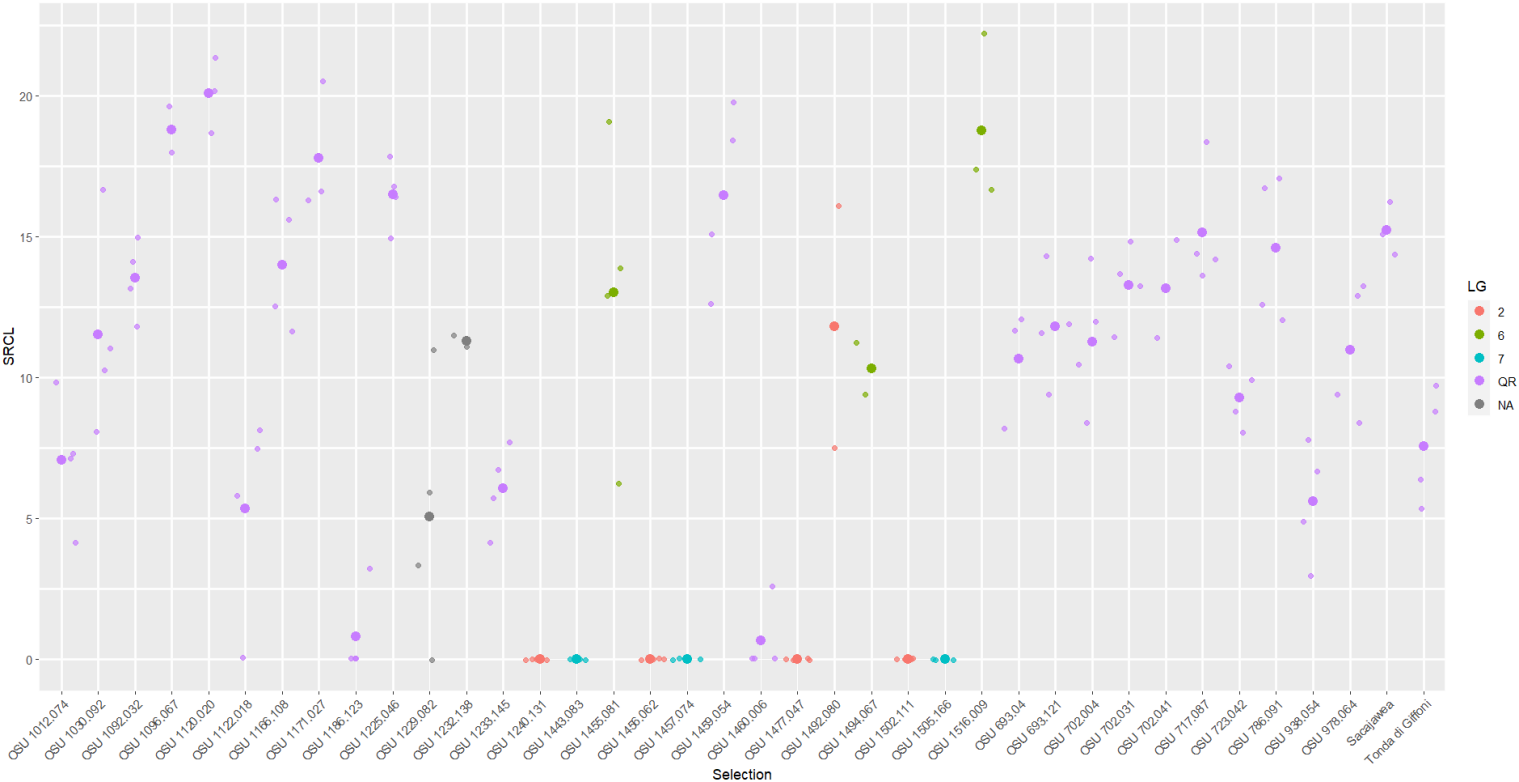
Scatter plot of square root total canker length (SRCL) measured on cultivars and selections in the 2017 replicated trial. Heavier dots signify cultivar/selection means while lighter dots signify values of individual trees. Colors denote linkage group or resistance source.

Selections related to *C. heterophylla* ‘Ogyoo’, ‘Crvenje’ from Serbia, ‘Uebov’ from Serbia, ‘Culplà from Spain, OSU 495.072 from southern Russia, and OSU 408.040 from Minnesota all developed extensive and, in many cases, fatal EFB, although some displayed a comparable SRCL and PDW to ‘Sacajawea’ at the completion of the study (**Table 3**; **Figure 1**). Two LG6 carrying offspring were evaluated in the 2017 experiment, OSU 1455.081 (OSU 408.040-related), and OSU 1494.067 (Russian 495.072-related), and displayed similarly severe disease, with each expressing more EFB than ‘Tonda di Giffoni’ (**Table 2**; **Figure 3**). OSU 1516.009 (*C. heterophylla* ‘Ogyoo’) was discovered as an escape of OSU EFB-screening during this trial based on ongoing observations in Corvallis, Oregon (S. Mehlenbacher, personal communication).

#### Resistance genes mapped to LG7

Selections carrying R genes mapped to LG7 were evaluated in the 2017 and 2019 experiments (**Table 1**). Three different resistance sources were tested in 2017 via selections OSU 1443.083 (*C. avellana* ‘Ratoli’), OSU 1505.116 (*C. americana* × *C. avellana* ‘Rush’) and OSU 1457.074 (*C. americana* hybrid ‘Yoder #5’ [a suspected offspring of ‘Rush’]), and each remained EFB-free through the duration of the experiment (**Tables 2**, **3**). In the 2019 experiment, resistance from ‘Ratoli’, ‘Rush, and ‘Yoder #5’ were again included as well as *C. avellana* OSU 1166.123 (Sochi, Russia). ‘Rush’ decedents included OSU 541.147 “The Beast” and its offspring OSU 1086.053 and OSU 1496.008. Combined, all three genotypes (n=30 trees) carrying the ‘Rush’ R-gene grew vigorously and developed no signs or symptoms of EFB for the duration of the trial. An offspring of ‘Yoder #5’, OSU 1062.055, also remained EFB-free throughout the 2019 trial, along with all 10 trees of OSU 1166.123 from Sochi, Russia (**Table 3**).

The 2019 experiment, however, harbored a striking development with EFB observed among four offspring of ‘Ratoli’ (OSU 1026.073, OSU 1382.092, OSU 1413.032, and OSU 1443.080). These data report the first known occurrence of ‘Ratoli’ R-gene carrying trees developing EFB (**Table 3**). Disease expression started in 2021 with a single, 9 cm long, canker on one tree of OSU 1413.032 but with all 39 other ‘Ratoli’ R-gene carrying trees remaining free of EFB that year. By 2023, that original single canker had grown to 39 cm long, and a new fully formed typical EFB canker appeared on a tree of OSU 1443.080 (located about 15 m from the first infected individual) and measured 40 cm long; all other ‘Ratoli’ R-gene carrying trees again showed no signs or symptoms of EFB in 2023. However, upon final evaluation in January 2024, fully formed EFB cankers were present on trees of all four selections in the 2019 trial, including 4 of 10 trees of OSU 1026.073, 5 of 10 trees of OSU 1382.092, and 1 of 10 (the original EFB infected tree) of OSU 1413.032, as well as 4 of 10 trees of OSU 1443.083. New EFB expression was primarily in the form of small (<30 cm) cankers with fully formed stromata (**Figures 2B, C**), that did not seem to affect tree growth, although both trees that expressed disease before 2024 (one tree each of OSU 1413.032 and OSU 1443.080) suffered canopy dieback above the original cankers. Infected individuals expressed mean PDW of 0.010 for OSU 1026.073, 0.016 for OSU 1382.092, 0.007 for OSU 1413.032, and 0.053 for OSU 1443.080 (**Table 3**).

No significant differences between ‘Ratoli’ R-gene carrying selections were detected for PDW, mean canker length, or SRCL. Interestingly, the four trees of ‘Ratoli’ offspring OSU 1443.083 that were planted in 2017 developed no signs or symptoms of EFB for the duration of the trial, after which they were moved via tree spade to a corner of the farm distant from the 2019 replicated trial, where they remain EFB free at the time of writing.

#### Resistance genes mapped to LG2

Eight selections carrying R-genes mapped to LG2 were evaluated in this study that originate from Turkey (Giresun) (n=2), the Republic of Georgia (n=5), and Holmskij, Russia (n=1) (**Table 1**). At the completion, only OSU 1492.080, an offspring of OSU 759.010 from the Republic of Georgia, succumbed to EFB. Its disease expression was severe, with PDW averaging 0.390 and 0.322 in the 2017 and 2019 trials, respectively, with limb and tree death observed in both trials (**Tables 2**, **3**). Interestingly, the other selections with resistance also derived from OSU 759.010 remained free of EFB and includes OSU 1440.026, OSU 1440.053, OSU 1456.062 and OSU 1477.047.

Selections from Turkey include OSU 1230.131 and OSU 1289.028. All trees of both remained free of disease. Russian (Holmskij #4) OSU 1168.013 also expressed no signs or symptoms of EFB. Trees of Turkish OSU 1230.131, and Georgian OSU 1456.062 and OSU 1477.047 were included in both the 2017 and 2019 trials.

#### Unmapped R-genes

Five selections from OSU suspected of carrying major R-genes that have not yet been mapped to a particular region on the hazelnut genetic map were also assessed for their EFB response. This includes OSU 1231.091 and OSU 1233.145 from Giresun, Turkey; OSU 1168.013 from Holmskij, Russia; OSU 1343.034, an offspring of the *C. colurna* × *C. avellana* hybrid “Gellatly Chinese trazel #11”, and OSU 889.084, an offspring of *C. americana* × *C. avellana* hybrid OSU 401.014 from Ohio.

Both Turkish selections, OSU 1231.091 and OSU 1233.145, expressed EFB, with all 10 trees of OSU 1231.091 developing relatively minor disease, with a mean PDW of 0.118, and only 1 tree of OSU 1233.145 developing EFB in the 2019 trial (**Table 3**). An additional four trees of OSU 1233.145 were evaluated in the 2017 experiment and all developed minor EFB symptoms (0.023 PDW) that were less severe than ‘Tonda di Giffoni’ (**Table 2**). The 10 trees of OSU 1168.013 from Holmskij, Russia developed no signs or symptoms of EFB for the duration of the trial. Additionally, trees of the *C. colurna* hybrid OSU 1343.034, also remained free of EFB. Finally, all ten trees of OSU 889.084 developed severe EFB with a mean PDW of 0.431, which was significantly higher (p=.003) than that of ‘Sacajawea’. All 10 trees had died from EFB by the completion of the study.

### Quantitative resistance to EFB

Two cultivars and 29 selections exhibiting QR in Oregon were evaluated for their EFB response in New Jersey in the 2017 trial (**Table 1**). ‘Sacajawea’ and ‘Tonda di Giffoni’ were included as controls in both trials as they had previously been characterized for EFB response in both locations. All four trees of ‘Tonda di Giffoni’ in the 2017 trial developed EFB (NC=3.5) expressing a SRCL of 33.9 √cm and 0.053 PDW. All trees of ‘Sacajawea’ in the 2017 trial expressed moderate QR, with a SRCL of 15.2 √cm, 0.167 PDW, and an average of 9.3 cankers per tree. Sufficient variation was observed across SRCL to support presence of variable disease responses between the cultivars, although means for NC and PDW were not significantly different (**Table 2**).

In the 2017 experiment, selections ranged widely in their QR response for each disease response trait (NC, SRCL, and PDW), and the greatest means separation was observed with SRCL (**Figures 3**, **2D**). Using the 0-5 scale, variation was consolidated with 19 of the selections expressing >3.5. SRCL ranged from 0.6 to 20.1 √cm. Two selections, OSU 1460.006 and OSU 1186.123, had a lower SRCL than ‘Tonda di Giffoni’, while four additional selections (OSU 1229.082 to OSU 1012.074; **Table 2**) had lower SRCL values than ‘Sacajawea’. Fifteen selections were not statistically different from ‘Tonda di Giffoni’ for SRCL (OSU 1229.082 to OSU 1166.108; **Table 2**), despite these selections ranging in SRCL from 9.3 to 14.0 √cm. The remaining seven selections are not significantly different from ‘Sacajawea’ (P=0.05) but range from 14.0 to 20.1 √cm. However, differences were observed between ‘Sacajawea’ and OSU 1096.067 and OSU 1120.020 for PDW, which was 0.775 and 0.535, respectively. Based on ranking of SRCL, 13 selections were between the QR control cultivars with values from 9.3 to 15.2 √cm, but there was not statistical support to separate these means in the current experiment.

## Discussion

Hazelnuts are a long-lived orchard crop that can remain commercially viable under proper conditions and management for well over 35 years. Chemical control of EFB is possible, but it is not always fully effective, and adds considerable costs to production (Julian et al., 2008). Consequently, there is a need for cultivars in North America to express durable forms of resistance to *A. anomala*, where an EFB-free or high QR phenotype is maintained over an orchard’s lifespan. This requirement is nested within a context of large production regions and high disease pressure in the Pacific Northwest, which is still home to many infected orchards, and new acreage expansion in the eastern regions where the pathogen is endemic and notably diverse (Muehlbauer et al, 2019; Tobia et al., 2024). There is an increased urgency for identifying durable resistance as exemplified in this study, as well as in recent developments in the Willamette Valley of Oregon, where OSU Extension has reported a new and aggressive EFB strain that is causing severe disease in orchards carrying the ‘Gasaway’ R-gene (Wiman, 2023). ‘Gasaway’ was the first reported resistance source and cultivars with this resistance have been widely planted and served an important role in revitalizing the U.S. industry. This study highlights many additional sources that are available that can support the development of cultivars expressing durable forms of resistance.

Many of the cultivars and selections carrying major R-genes, all of which expressed resistance to EFB at OSU at the initiation of this study, showed variable responses when exposed to EFB in New Jersey. Notably, those carrying R-genes mapped to LG6 developed severe disease symptoms that were most often worse than ‘Sacajawea’ and fatal. Importantly, however, the two selections OSU 1390.008 and OSU 1352.059 (offspring of Farris 533.029) with R-genes that also map to LG6, and whose resistant phenotype was maintained, suggest potential for allelic variation and/or different resistance loci within a gene cluster in the region. A similar scenario may also present itself in resistance regions mapped to other LGs.

Case in point, this study is the first reported EFB incidence on trees carrying the ‘Ratoli’ R-gene (LG7), or any LG7 source, which had previously held up to EFB in New Jersey for 20+ years (Capik and Molnar, 2012, Molnar unpublished). By the conclusion of the study, EFB had also been identified in other nearby plots at Rutgers on ‘Ratoli’ related trees that had persisted there EFB-free for over a decade (Molnar, unpublished) – indicating a distinct shift in EFB resistance expression. While infections on ‘Ratoli’ offspring were generally minor, and canopy dieback was only identified in two individuals, EFB cankers developed on all Ratoli-related selections (n=4) in the 2019 experiment. Results showed disease on LG7 sources remained isolated only to those with the ‘Ratoli’ lineage, however. The six selections carrying the *C. americana* ‘Rush’ R-gene, and one selection, OSU 1166.123 of Sochi, Russia, remained free of EFB and suggest, like with Farris 533.029, that allelic diversity may also exist in the LG7 resistance region. Offspring of ‘Rush’ have been planted in the eastern U.S. for over 100 years, albeit not under the high acreage of the Willamette Valley, but these cultivars nevertheless offer a promising outlook for the role major R genes may still play in breeding durable resistance.

Selections with resistance mapped to LG2, including multiple distinct sources from Turkey, Russia (Holmskij), and the Republic of Georgia, maintained their EFB-free status. The exception was OSU 1492.080, which was represented by 14 trees between the 2017 and 2019 experiments. It consistently developed severe EFB resembling a fully susceptible phenotype. Interestingly, all the other selections (n=5) that share the same grandparent R-gene donor (OSU 759.010) with OSU 1492.080 remained free of EFB. For these reasons, OSU 1492.080 is suspected to be a susceptible escape from OSU screenings that does not in fact carry the OSU 759.010 R-gene.

In this study, we also identified dozens of selections that maintained moderate to high levels of tolerance to EFB in New Jersey. The variation in EFB response across QR selections was wide, from no EFB, to highly tolerant, to moderately tolerant to susceptible (**Figure 2D**), supporting the premise that EFB QR in hazelnut is controlled in a multi-genic fashion. Further, the EFB response of the QR selections contrasts with most major R-gene carrying selections, where in general, they expressed only a binary pattern of no EFB or severe EFB. Notably, high levels of QR comparable to ‘Sacajawea’ and ‘Tonda di Giffoni’ were verified in selections from diverse origins, including Armenia, Azerbaijan, and Turkey. These selections represent potential opportunities to diversify QR breeding populations, although heritability studies are needed. While only a few QR selections expressed resistance/tolerance levels required for cultivar release, most demonstrate QR equivalent to or greater than ‘Sacajawea’, which has demonstrated its significant value as a QR donor parent in the recent EFB-resistant releases ‘Hunterdon’ and ‘Monmouth’ (Molnar, 2022). Moderate levels of QR can combine in additive fashion to yield transgressive segregants with higher levels of QR (Osterbauer et al., 1997). The additive effect of some QR × QR crosses is exemplified by breeding at Rutgers. ‘Raritan’ was selected from a cross of ‘Sant Pere’ offspring OSU 539.031 and ‘Tonda di Giffoni offspring OSU 616.018. ‘Raritan’ rarely exhibits any signs or symptoms of EFB in New Jersey (Molnar et al, 2020; US Plant Patent #32,460P2).

While our data supports the instability and fatality of some R-gene sources over time (or between environments, i.e., Oregon versus New Jersey), QR selection performance is relatively stable in that most express a disease response in between that of the control cultivars rather than significantly less. These findings suggest that QR may serve an important role in breeding for durable resistance or in support of cultivation under pressure from different *A. anomala* populations. QR can support enhanced durability through QR × QR breeding schemes, as described above, or QR × R-gene schemes, where the quantitative genetic background prolongs an R-gene’s viability and provides a “safety net” should the major gene fail (Pilet-Nayel et al., 2017)). While this strategy has clear appeal, successfully carrying out such a crossing scheme requires DNA markers or genomic prediction models to support the selection of offspring with QR QTL, as the QR phenotype will be masked in progeny co-inheriting the R-gene. An additional promising scheme includes R-gene pyramiding (R × R) although limitations are arising with some LG6 and now LG7 R-gene sources being overcome. Nevertheless, there remains a positive outlook with some sources at these LGs still providing protection against the diverse strains in New Jersey. New discoveries of major gene resistance recently reported on LG1 and LG4 from *C. americana* hybrids OUS 401.014 and OSU 1044.086 (Heilsnis et al., 2024; Mooneyham et al., 2024), respectively, support the presence of a wider diversity of resistance regions that may also be available for continued efforts.

## Conclusion

The variable response between plant materials evaluated in Oregon and New Jersey, and associations between LGs and disease expression clearly demonstrate the existence of *A. anomala* populations or strains capable of overcoming major R-gene resistance. When combined with the long-term nature of hazelnut orchards, it is clear that breeding and research efforts aimed at elucidating and developing durable resistance to *A. anomala* should be pursued. Specifically, additional studies are needed to reproduce infections on major R-gene carriers in controlled conditions to verify pathogenic variation between isolates of *A. anomala*. Genomic and gene expression tools can be utilized to identify genetic changes in the pathogen populations that have overcome R genes, and to help identify/characterize R-genes and potential *A. anomala* races to facilitate disease management. Fortunately, this study identifies dozens of unique sources of resistance and tolerance to EFB showing effectiveness in New Jersey and Oregon, constituting a diverse genetic base for future breeding efforts that can include R gene pyramiding, and/or the use of QR and interspecific hybridization.

## Data availability statement

The raw data supporting the conclusions of this article will be made available by the authors, without undue reservation.

## Author contributions

DJ: Data curation, Formal analysis, Methodology, Writing – original draft, Writing – review & editing. RR: Formal analysis, Investigation, Methodology, Visualization, Writing – review & editing. JC: Methodology, Writing – review & editing. SM: Conceptualization, Resources, Writing – review & editing. TM: Conceptualization, Data curation, Funding acquisition, Investigation, Methodology, Project administration, Resources, Supervision, Writing – original draft, Writing – review & editing.
